# Supplementary material for: Splice-Junction-Based Mapping of Alternative Isoforms in the Human Proteome
Source: Cell Rep. Author manuscript; Available in PMC 2020 Jan 15. (PMC6961840; doi:10.1016/j.celrep.2019.11.026)
Supplement: 2 [file NIHMS1546469-supplement-2.zip › DF1/PXD010154/Heart/Heart_71_MAF1_GDLPGEAINSQLTVETGDAHIIGR.pdf]

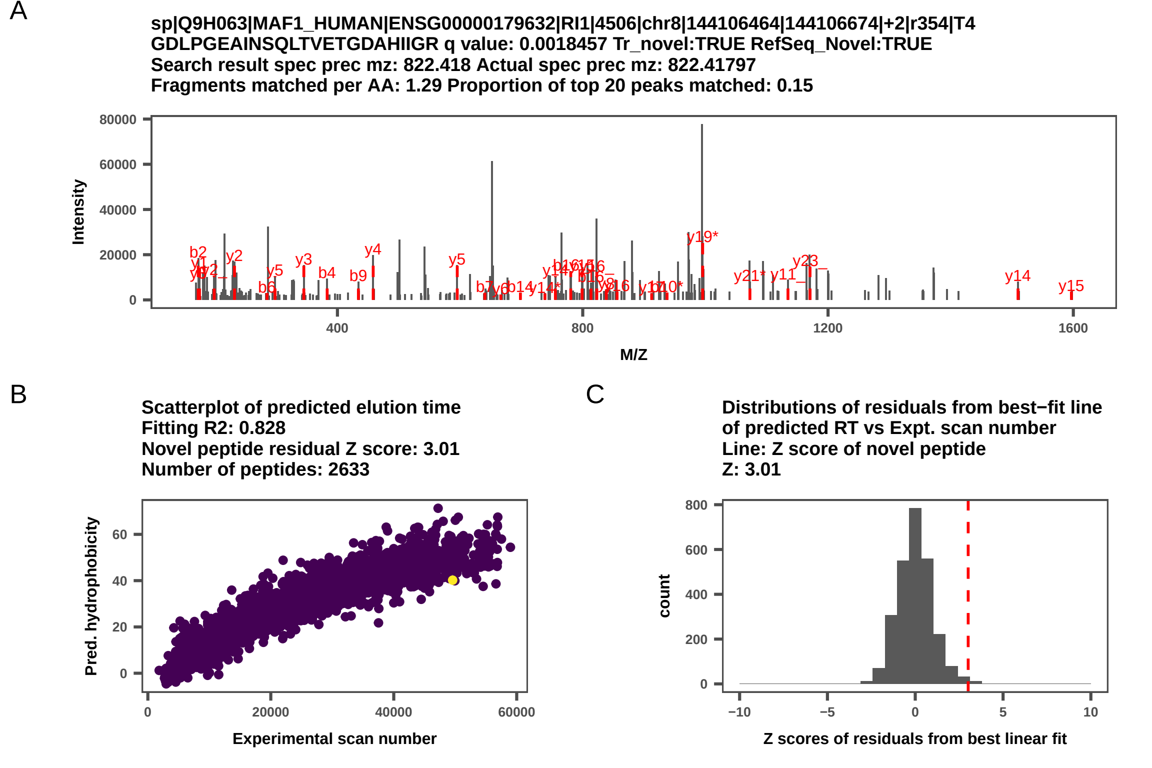
A
sp|Q9H063|MAF1_HUMAN|ENSG00000179632|RI1|4506|chr8|144106464|144106674|+2|r354|T4
GDLPGEAINSQLTVETGDAHIIGR q value: 0.0018457 Tr_novel:TRUE RefSeq_Novel:TRUE
Search result spec prec mz: 822.418 Actual spec prec mz: 822.41797
Fragments matched per AA: 1.29 Proportion of top 20 peaks matched: 0.15
Intensity
80000
60000
40000
20000
0
b2
y2
y3
y4
y5
b4
b9
b6
y19*
y21*
y14
y15
400
800
1200
1600
M/Z
B
Scatterplot of predicted elution time
Fitting R2: 0.828
Novel peptide residual Z score: 3.01
Number of peptides: 2633
Pred. hydrophobicity
60
40
20
0
0
20000
40000
60000
Experimental scan number
C
Distributions of residuals from best−fit line
of predicted RT vs Expt. scan number
Line: Z score of novel peptide
Z: 3.01
count
800
600
400
200
0
−10
−5
0
5
10
Z scores of residuals from best linear fit
